# Allopurinol Resistance in *Leishmania infantum* from Dogs with Disease Relapse

**DOI:** 10.1371/journal.pntd.0004341

**Published:** 2016-01-06

**Authors:** Daniel Yasur-Landau, Charles L. Jaffe, Lior David, Gad Baneth

**Affiliations:** 1 Koret School of Veterinary Medicine, The Hebrew University, Rehovot, Israel; 2 Department of Microbiology and Molecular Genetics, IMRIC, Hadassah Medical School, The Hebrew University, Jerusalem, Israel; 3 Department of Animal Sciences, The Hebrew University, Rehovot, Israel; Universidad Autónoma de Yucatán, MEXICO

## Abstract

**Background:**

Visceral leishmaniasis caused by the protozoan *Leishmania infantum* is a zoonotic, life threatening parasitic disease. Domestic dogs are the main peridomestic reservoir, and allopurinol is the most frequently used drug for the control of infection, alone or in combination with other drugs. Resistance of *Leishmania* strains from dogs to allopurinol has not been described before in clinical studies.

**Methodology/Principal Findings:**

Following our observation of clinical disease relapse in dogs under allopurinol treatment, we tested susceptibility to allopurinol of *L*. *infantum* isolated from groups of dogs pre-treatment, treated in remission, and with disease relapse during treatment. Promastigote isolates obtained from four treated relapsed dogs (TR group) showed an average half maximal inhibitory concentration (IC50) of 996 μg/mL. A significantly lower IC50 (*P* = 0.01) was found for isolates from ten dogs before treatment (NT group, 200 μg/mL), as well as for five isolates obtained from treated dogs in remission (TA group, 268 μg/mL). Axenic amastigotes produced from isolates of the TR group also showed significantly higher (*P* = 0.002) IC50 compared to the NT group (1678 and 671 μg/mL, respectively). The lower sensitivity of intracellular amastigotes from the TR group relative to those from the NT group (*P* = 0.002) was confirmed using an infected macrophage model (6.3% and 20% growth inhibition, respectively at 300 μg/mL allopurinol).

**Conclusions:**

This is the first study to demonstrate allopurinol resistance in *L*. *infantum* and to associate it with disease relapse in the canine host. These findings are of concern as allopurinol is the main drug used for long term control of the disease in dogs, and resistant *L*. *infantum* strains may enhance uncontrolled transmission to humans and to other dogs.

## Author Summary

Visceral leishmaniasis caused by *Leishmania infantum* is a widespread, devastating zoonotic disease. Domestic dogs are considered the major reservoir for human *L*. *infantum* infection in an area stretching from Portugal to China, and across South America. Dogs not only carry infection and transfer it to humans via sand flies, but frequently suffer from a severe and fatal disease. Allopurinol is the main drug used for treatment of dogs with leishmaniasis, by itself or in conjunction with other drugs. Following treatment, most dogs experience clinical remission, but this is often not followed by parasite elimination. We found that dogs suffer from disease relapse during treatment with allopurinol, and showed that *L*. *infantum* promastigotes isolated from treated relapsed dogs were roughly four times less susceptible to allopurinol as compared to isolates from dogs before treatment or from dogs that resolved their clinical disease during treatment. We also confirmed that resistance to allopurinol was present in axenic and intracellular amastigotes. This study describes resistance of *L*. *infantum* clinical isolates associated with disease relapse. Allopurinol resistance may increase *L*. *infantum* transmission to humans and to other dogs, as canines with uncontrolled infection are highly infectious to sandflies.

## Introduction

Visceral leishmaniasis caused by *L*. *infantum* is a potentially fatal disease, which is a serious public health concern in Europe, Asia, North Africa and Latin America. The dog is the main reservoir for this zoonotic infection and it has been estimated that in Southwestern Europe alone there are about 2.5 million infected dogs [1,2].

Allopurinol is a purine analog used mainly as a xanthine oxidase inhibitor to reduce serum urate concentration and is prescribed for the management of gout in humans [3]. Allopurinol’s anti-leishmanial activity was first described in 1974 by Pfaller and Marr [4], and is attributed to the inhibition of the leishmanial enzyme hypoxanthine-guanine phosphoribosyl transferase (HGPRT). HGPRT takes part in the parasite's purine salvage pathway, converting dephosphorylated purines to nucleoside monophosphates. Phosphorylated allopurinol is most likely incorporated into nucleic acids, leading to disrupted protein translation and selective parasite death [5,6]. Allopurinol has limited use as an agent for treatment of human visceral and cutaneous leishmaniasis, and it was rarely administered in conjunction with amphotericin B or pentavalent antimonials [1,7]. In veterinary medicine however, it is considered the major first line drug for long term treatment of canine leishmaniasis (CanL), often in combination with pentavalent antimonials or miltefosine for the first month and then continued alone [8,9]. Among its merits are wide availability, low cost, good safety profile, lack of known resistance, and the fact that it is rarely used for the treatment of human leishmaniasis. For the latter reason, allopurinol is the only drug recommended by the World Health Organization for the treatment of CanL [1]. Since antimonials and miltefosine are not commercially available for treatment of CanL in Israel, allopurinol is currently the only drug used against this disease in dogs.

Long term allopurinol treatment, whether combined with an initial course of meglumine antimoniate or miltefosine [8,10,11], or administered alone [12,13], results in most cases in improvement of the dog's clinical disease signs within a few weeks and a reduction in the dog's infective potential. Despite that, cases of disease relapse following secession of combined or exclusive allopurinol treatment [14,15,16] or even during treatment [17,18] have been recorded. Despite a growing concern over the development of resistance to other antileishmanial drugs in humans and increased efforts to uncover the genetic and biochemical basis of resistance mechanisms [19,20,21,22,23,24], very little information is available on resistance to anti-leishmanial drugs in dogs and no direct association has been documented between disease relapse and drug resistance [25,26,27]. In this study, following our observation of clinical disease relapse in dogs under allopurinol treatment, we tested susceptibility to allopurinol of *L*. *infantum* strains isolated from relapsed and non-relapsed dogs aiming to explore the possibility of drug resistance.

## Methods

### Dogs

Nineteen privately owned dogs with natural *L*. *infantum* infection were included in the study. The inclusion criteria were: diagnosis of leishmaniasis as detailed under “diagnosis of leishmaniasis”, documented medical history from diagnosis, no concurrent non-*Leishmania* related medical conditions and owners consent to participate in the study. Dogs were allocated to one of three groups, based on a clinical score [28] established for each dog on its enrollment to the study: (1) ten newly diagnosed dogs with moderate to severe clinical signs (clinical score >2) due to *L*. *infantum* infection, prior to initiation of allopurinol treatment (non-treated; NT); (2) five previously diagnosed dogs with clinical disease undergoing allopurinol treatment and currently in remission for 3 months or longer (clinical score >2) (treated-asymptomatic; TA); and (3) four previously diagnosed dogs undergoing allopurinol treatment, with recurrence of clinical signs (clinical score >2) after being disease free for 3 months or longer (treated-relapsed; TR). Dogs belonging to the TA and TR groups were treated with allopurinol at 10 mg/kg orally every 12 hours from diagnosis onward, with no intermissions. None of the dogs experienced any previous episodes of leishmaniasis or was treated with allopurinol for any indication before the described diagnosis, nor did they receive any other anti-leishmanial drug at any stage. For dogs in the TR and TA groups, clinical score was established for time of disease diagnosis based on information in their medical records. Complete blood count, albumin, total protein, creatinine and urea were measured for each dog on enrollment to the study.

### Diagnosis of leishmaniasis

Diagnosis of leishmaniasis was made in all dogs based on the presence of compatible clinical signs, positive ELISA serology, and parasite isolation in culture followed by molecular characterization. Amplification of *L*. *infantum* DNA from blood by PCR was attempted for each patient. Serology was tested on crude *L*. *infantum* antigen ELISA as previously described [29] with a cutoff value set at 0.6 optical density (OD). Real time PCR was used to detect and quantify *Leishmania* DNA in blood. DNA was extracted (illustra, GE Healthcare, UK) from 200μL blood and a 120 bases long leishmanial kDNA fragment was amplified by PCR using primers JW11 (5-CCTATTTTACACCAACCCCCAGT-3) and JW12 (5-GGGTAGGGGCGTTC-TGCGAAA-3) [30]. Reactions were done in a total volume of 20μL, including; 10 μL Fast SYBR Green Master Mix (x2) (Applied Biosystems, Foster City, CA), 1μL DNA, ultra-pure water and a final concentration of 500nM of the forward and reverse primers. Reactions were carried out using the StepOnePlus real-time PCR thermal cycler (Applied Biosystems, Foster City, CA) with the following thermal profile; initial denaturation for 20 seconds at 95°C, followed by denaturation for 3 seconds at 95°C and annealing for 30 seconds at 59°C for 40 cycles. Amplicons were subsequently subjected to a melt step with the temperature raised to 95°C for 15 seconds and then lowered to 60°C for 1 min. The temperature was then raised to 95°C at a rate of 0.3°C per second. Amplification, melt profiles and quantity based on analysis of standard curve values, were analyzed using the StepOne V2.2.2 software (Applied Biosystems, Foster City, CA). Samples were tested in duplicates including negative (*Leishmania*-free dog DNA) and positive *L*. *infantum* (MCAN/IL/2002/Skoshi) controls. Only samples with CT value below 35 and a melt curve matching that of the positive control were considered positive. Quantitation of leishmanial DNA in the samples was made by comparing samples to a calibration curve. *Leishmania* DNA standards representing 10^0^−10^6^ parasites DNA per μL were produced by mixing 200μL of *Leishmania* free dog blood with 2*10^2^−2*10^8^ cultured *L*. *infantum* promastigotes, followed by DNA extraction as described above.

### Parasite isolation and culture as promastigotes and axenic amastigotes

Parasites were isolated from popliteal or prescapular lymph nodes of all dogs by needle aspiration prior to initiation of treatment (NT group) or during treatment (TA and TR groups). Samples were aspirated aseptically and seeded in semi-solid medium [31] for 5–10 days at 27°C. Viable promastigotes were transferred to complete medium 199 (M-199) adjusted to pH 6.8 [32]. Promastigotes were tested for allopurinol susceptibility after 5–10 passages.

Axenic amastigotes were generated from promastigote isolates as described by Sereno and Lemesre [33], with the following modifications; M-199 was complemented as described above [32] only with 20% FCS, pH was adjusted to 5.8 with sodium phosphate buffer and 5x10^6^ logarithmic phase promastigotes per mL from each tested isolate were used for induction of differentiation. Axenic amastigotes were kept at 37°C and passaged thrice to ensure viability before testing.

### Parasite molecular identification

DNA from promastigote isolates was extracted (illustra, GE Healthcare, UK) and a 300 bases long leishmanial ITS1 fragment was amplified by PCR using the primers LITSR (5'-CTGGATCATTTTCCGATG-3') and L5.8S (5'-TGATACCACTTATCG-CACTT-3') as previously described [34]. Amplicons were sent for DNA sequencing and the resulting sequences compared to *L*. *infantum* sequences deposited in GenBank using the BLAST program (www.ncbi.nlm.nih.gov/BLAST).

### Promastigote growth curves

Growth curves were made for three isolates from the NT and TR groups each, in order to determine whether exposure to allopurinol affected the isolates’ growth kinetics. Samples containing 1x10^6^ per mL stationary phase promastigotes were transferred into complete medium. Promastigote concentrations were determined for each isolate every 24 hours for six consecutive days in triplicates using a Neubauer counting chamber. Average daily values were plotted, and the experiment was repeated twice for each isolate.

### Allopurinol IC50 determination for promastigotes and axenic amastigotes

Half-maximal inhibitory concentration (IC50) values were determined by a viability-based assay as described [32] with modifications. For each isolate, 1x10^6^ log phase promastigotes or axenic amastigotes were aliquoted in triplicates (100μL/well) into 96-well flat-bottom plates (Nunc, Denmark). Allopurinol (Sigma-Aldrich, St. Louis, MO) from a stock solution of 50mg/mL in 1N NaOH was 1:2 serially diluted in complete medium and added (100μL/well) to final concentrations of 0–1600 μg/mL. Three wells per plate containing complete medium were used as blanks. Plates were incubated for 66 hours, followed by addition of 20μL resazurin (alamarBlue, AbD Serotec, UK) per well. Plates were incubated for a further 6 hours, and fluorescence (λ_ex_ = 540nm, λ_em_ = 590nm) was read using a fluorescent microplate reader (Synergy2, BioTek Instruments, Winsooki, VT). Data was analyzed using the Prism 5 software (GraphPad Software, San Diego, CA). Experiments were repeated twice for each isolate tested, 7–14 days apart, and the average IC50 value for each isolate and stage was used in further analysis.

### Toxicity of allopurinol for macrophage cell lines and allopurinol susceptibility of intracellular amastigotes

The cytotoxicity of allopurinol for five different macrophage cell lines was first determined in order to identify the best host cell line for testing drug activity on intracellular amastigotes. Cytotoxicity IC50 was measured, essentially as described above for the parasites, with some modifications. Human THP-1 and U-937 (provided by Prof. Charles Jaffe’s laboratory, Hebrew University), murine RAW 264.7 (kindly provided by Professor Nachum Shpigel’s laboratory, Hebrew University) and J774.1, and canine DH-82 cell lines (the latter 2 cell lines kindly provided by Prof. Shimon Harrus’s laboratory, Hebrew University) were cultured in the following media: RPMI for THP-1 and U-937, DMEM for RAW 264.7 and J774.1, and MEM for DH-82; all supplemented with 10% FCS, 2mM L-glutamine and antibiotics (100IU penicillin G and 100μg/mL streptomycin). For each cell line, 1x10^4^ cells were aliquoted in triplicates into 96-well plates. Allopurinol was added to final concentrations of 0, 12.5, 25, 50, 100, 200, and 400μg/mL. Cells were incubated for 66 hours at 37°C, 20μL resazurin were added and cultures were further incubated for an additional 4 hours. Two separate experiments were performed for each cell line and the average IC50 value was used for analyses.

Allopurinol susceptibility of intracellular amastigotes was evaluated using isolates from the NT and TR groups. DH-82 cells in complete MEM medium, chosen based on the cytotoxicity study above, were plated (1x10^4^ cells/0.5mL/well in duplicates) on round glass slides in 24 well plates (Nunc, Denmark) and left for 6 hours to adhere. Promastigotes from a stationary culture of the test isolate were then added for 16 hours at 37°C using an infection ratio of 10:1 parasites:macrophage. Cells were washed twice with MEM without FCS to remove extracellular parasites, and 1mL complete MEM containing allopurinol added (final concentration being either 0 or 300μg/mL). Cells were incubated for 72 hours, washed twice with PBS, followed by fixation in methanol and Giemsa staining of the glass slides. Intracellular parasites for each isolate were counted in 100 host cells, and percent amastigote growth inhibition was calculated compared to the non-treated controls. Each experiment was repeated twice and the average percent growth inhibition was calculated.

### Statistical analysis

Significance of differences in IC50’s, as well as other quantitative variables such as clinical score, serology OD or blood and biochemistry indices among the three study groups was determined using the non-parametric Kruskal-Wallis test. The Mann-Whitney non-parametric test was used to compare IC50’s between two independent groups and for multiple pairwise comparisons (as a Post Hoc test, with the Bonferroni correction of the significance level). The Fisher's exact test was applied to examine the association between two categorical variables. Association between IC50 and the serology OD or treatment duration was estimated by calculation of the Pearson and the non-parametric Spearman rank correlation coefficients. Assessing the difference in growth rates between promastigotes from two study groups was done by applying the repeated measures ANOVA model, with the Greenhouse-Geiser test for effects within each group. Comparison of clinical scores at two time point within a group was done for the TR and TA groups using the Wilcoxon Signed Ranks Test. All statistical tests applied were two-tailed, and a *P*-value of 5 percent or less was considered statistically significant. Average values are given with the respective standard deviation. Analysis was made using the PASW 18 software (SPSS Inc., Chicago, IL).

### Ethics statement

Procedures to identify *Leishmania* infection in the dogs and medical treatment were carried out as a part of their routine management with informed consent of the dog’s owners, and in compliance with the Hebrew University's animal use ethical guidelines. The animal care protocol was approved by the Hebrew University’s Institutional Animal Care and Use Committee (IACUC) which adheres to the NIH guidelines; approval no. MD-08-11476-2.

## Results

### Dogs

The 19 parasite strains included in this study were derived from 10 non-treated dogs (NT), and from 9 dogs undergoing allopurinol treatment including five dogs that have gone into remission following treatment (TA) and 4 relapsed (TR). Complete information of each dog and respective parasite strain can be found in Tables 1 and S2.

**Table 1:** Characteristics and test results of infected dogs included in the study, and codes for *Leishmania infantum* strains isolated from them.

	Gender	Age (y)	Treatment duration (mo)^a^	Clinical score at diagnosis	Clinical score at enrollment	ELISA serology (OD)	Blood kDNA PCR	Isolate ID
Non- treated (NT)								
	F	3	-	^c^	3	1.74	+	NT1
	M	8	-		7	1.05	+	NT2
	F	1.5	-		3	1.57	+	NT3
	M	11	-		6	1.62	+	NT4
	M	5	-		4	1.60	+	NT5
	M	11	-		4	1.11	+	NT6
	M	11	-		6	0.94	+	NT7
	M	2.5	-		7	1.51	+	NT8
	F	5.5	-		5	1.79	+	NT9
	F	5	-		5	1.27	-	NT10
	Mean	6.4±3.7			5±1.5	1.42±0.3		
Treated asymptomatic (TA)								
	M	5	11	5	1	0.84	-	TA1
	F	3	10	6	0	1.13	-	TA2
	M	11	4	5	1	1.25	-	TA3
	F	6	7.5	3	1	1.34	-	TA4
	F	6	19	5	2	0.98	-	TA5
	Mean	6.2±2.9	10.3±5.6	4.8±1.1	1±0	1.11±0.2		
Treated relapsed (TR)								
	F	6	36	4	5	0.51	-	TR1
	F	8	36	6	7	1.28	+	TR2
	F	10	24	4	5	1.78	ND^b^	TR3
	F	3	4	4	6	1.54	-	TR4
Mean		6.8±3	25±15.1	4.5±1.0	5.8±1.0	1.28±0.6		

^a^ Duration of allopurinol treatment determined as time from start of treatment to parasite isolation during treatment.

^b^ ND—not done.

^c^ diagnosis and enrolment describe one time point in the NT group, therefore the same clinical scores were used for both. Means are presented ± STDEV.

The ages of infected dogs varied between 1.5 and 11 years, with mean of 6.4±3.7, 6.2±2.9 and 6.8±3.0 years for the NT, TA and TR groups, respectively. No significant difference was found between the average ages of the three groups (Kruskal-Wallis test, *P* = 0.92). Eleven females and eight males were included in the study groups. No significant difference in gender distribution was found between the study groups (Fisher's Exact test, *P* = 0.154), even though females were over-represented in the TR group.

All dogs presented clinical signs compatible with CanL at diagnosis, and in group TR also at enrollment (for group NT diagnosis and enrollment were at the same time point) [8]. The average clinical score at disease diagnosis was 4.5±1.0, 4.8±1.1 and 5±1.5 for the TR, TA and NT groups, respectively, with no significant difference between the groups (Kruskal-Wallis Test, *P* = 0.79). The clinical score for the TA group was significantly lower on enrollment, both compared to group’s average score at diagnosis (1±0 versus 4.8±1.1, Wilcoxon Signed Ranks Test, *P* = 0.042) and the score at enrollment for the NT and TR groups (Mann-Whitney Test, *P* = 0.001 and 0.016, respectively). No significant differences were found in blood and biochemistry indices between groups, except for a higher total protein for the NT group compared to the TA group (Mann-Whitney Test, *P* = 0.028).

The average duration of treatment was 10.3±5.6 and 25±15.1 months for the TA and TR groups, respectively, with no significant difference between groups (Mann-Whitney test, *P* = 0.19). Nine out of ten dogs in the NT group were positive for *L*. *infantum* by blood PCR, compared to one out of eight of tested treated dogs (groups TA and TR). The average ELISA serology OD was 1.42±0.3, 1.11±0.2 and 1.28±0.6 for the NT, TA and TR groups, respectively, with no significant difference between groups (Kruskal-Wallis test, *P* = 0.256).

### Parasite identification and growth kinetics

**Fig 1:**
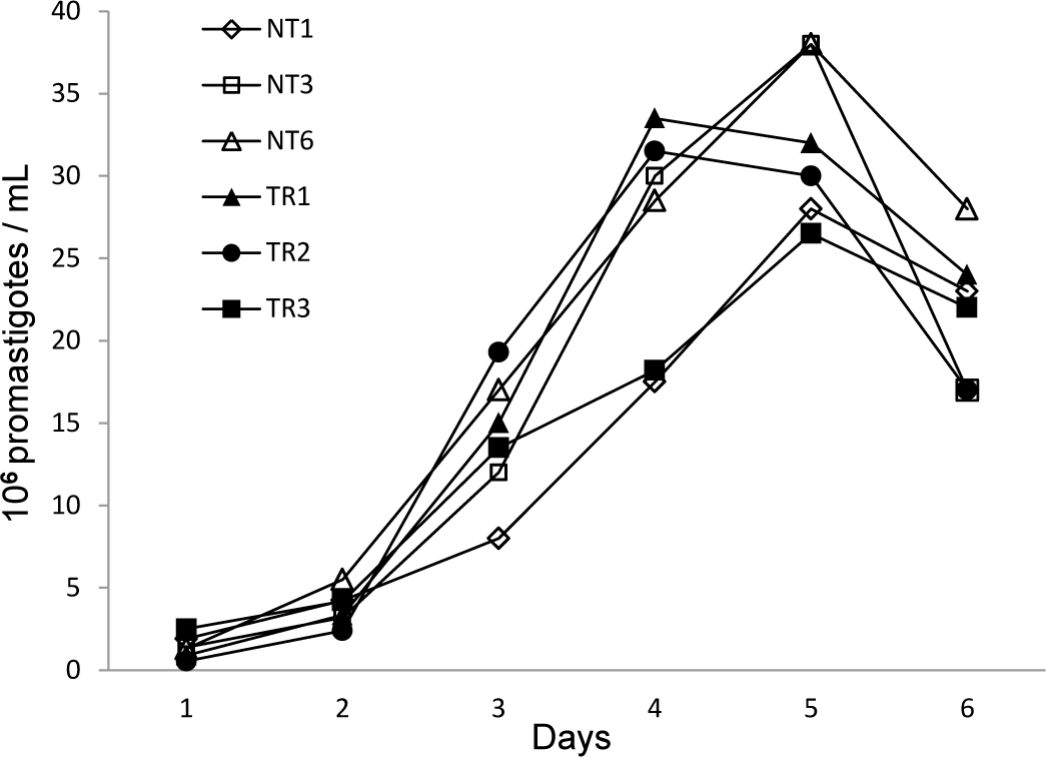
Promastigote growth curves of strains from the non-treated (NT) and treated relapsed (TR) groups. No significant differences were found between promastigote counts for any of the isolates on respective days (Repeated measures ANOVA with Greenhouse-Geisser tests, *P =* 0.784).

All of the isolates obtained from dogs were identified as *L*. *infantum* with 99–100% similarity by BLAST to *L*. *infantum* ITS1 sequences GI339730635, GI332330750, GI306422161 and deposited in GenBank (S1 Table; GenBank accessions KM677128- KM677146). Comparison of promastigote growth curves of 3 isolates from the NT and TR groups each demonstrated that all tested strains entered the logarithmic growth phase after 2 days in culture (Fig 1). Stationary phase was achieved after an average of 4.67±0.6 or 4.33±0.6 days for the NT and TR strains, respectively. No significant difference was found between promastigote counts for strains from the two groups on any of the days (Repeated measures ANOVA with Greenhouse-Geisser tests, *P =* 0.784). Based on this data we could conclude that there was no inherent difference in growth kinetics between naïve isolates and those previously exposed to allopurinol, thus we proceeded to test the IC50 using similar incubation times for all isolates.

### Toxicity of allopurinol to macrophage cell lines

Allopurinol toxicity for 5 different cell lines, expressed as allopurinol IC50 value for each cell line, was determined (Table 2). The canine macrophage cell line, DH-82, was found to have the highest IC50, 377±3.25μg/mL. Because the other cell lines showed IC50’s 1.6–5.3 folds lower, the DH-82 cell line was chosen for evaluating the susceptibility of intracellular amastigotes to allopurinol. The allopurinol concentration chosen for this assay was 300μg/mL, which caused approximately 20% growth inhibition of the DH-82 cells in the absence of parasites.

**Table 2:** Allopurinol toxicity for cell lines tested in this study.

Cell line	Origin	Allopurinol IC50 μg/mL (Mean±STDEV)
U-937	Human - histiocytic lymphoma	81.8±6.0
THP-1	Human—acute monocytic leukemia	142.1±11.7
RAW 264.7	Murine—Abelson murine leukemia virus-induced tumor	71.0±3.2
J774.1	Murine—reticulum cell sarcoma	233.0±0.1
DH-82	Canine—malignant histiocytosis	377.2±32.5

### Allopurinol susceptibility of promastigotes, axenic amastigotes and intracellular amastigotes

**Fig 2:**
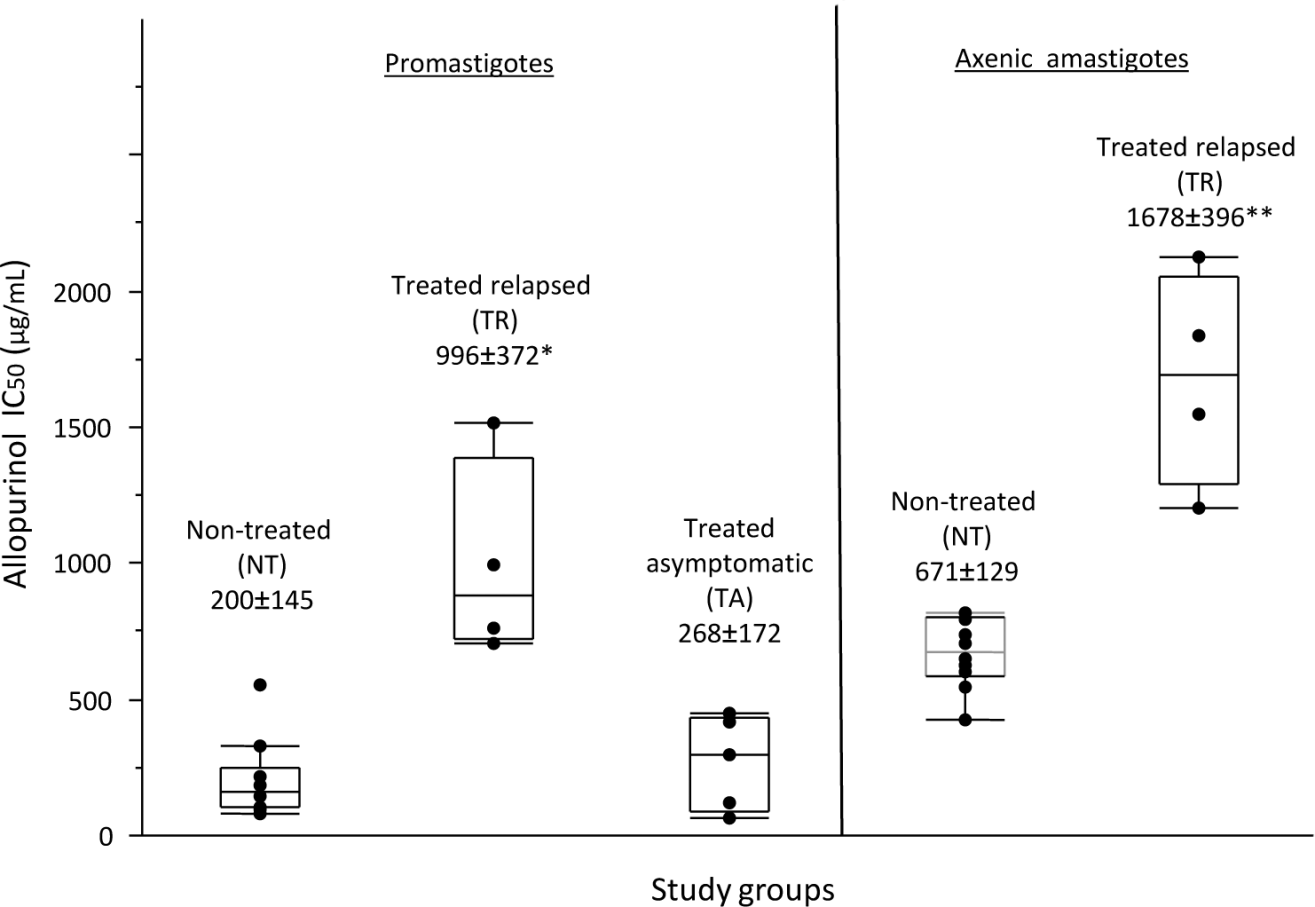
Average allopurinol IC50 values of promastigote and axenic amastigotes. Average allopurinol IC50 values found for promastigotes and axenic amastigotes of isolates from dogs in the study groups. Numbers in labels represent group mean ± STDEV. The TR group was found to have average IC50 values 5 and 2.5 times higher than the NT group for promastigotes and axenic amastigotes, respectively. * Represents significant difference between promastigote groups (*P* = 0.01). ** Represents significant difference between axenic amastigote groups (*P* = 0.002).

All isolates (n = 19) were tested as promastigotes. The average IC50 found for promastigotes from the TR group was approximately fourfold higher (996±372 μg/mL), and significantly different from that of the two other groups (Kruskal-Wallis test, *P* = 0.01) (Fig 2 and S2 Table). No significant difference was noted between the average allopurinol IC50 for promastigotes in the NT group (200±145μg/mL) compared to the TA group (268±172μg/mL) (Mann-Whitney test, *P* = 0.594). Sensitivity of axenic and intracellular amastigotes to allopurinol was determined for parasite strains of the NT and TR groups (n = 10 and 4, respectively). The IC50 observed for the axenic amastigote stage (Fig 2 and S2 Table) was consistently higher, by 1.5 to 10.2 fold compared to the corresponding promastigote stage of each strain, regardless of the treatment history of the dog. As seen for promastigotes, average allopurinol IC50 value for axenic amastigotes from group TR (1678±396 μg/mL) was also more than two folds higher than this found for isolates from group NT (671±129μg/mL, Mann-Whitney test, *P* = 0.002). Finally, allopurinol at 300μg/mL resulted in an average 20±4.9 percent growth inhibition for NT group intracellular amastigotes in DH-82 infected macrophages, which was significantly higher compared to 6.3±4.8 percent growth inhibition for the TR group isolates (Mann-Whitney test, *P* = 0.002) (Tables 3 and S2). We were unable to use higher allopurinol concentrations in order to determine the exact IC50 value for intracellular amastigotes, since concentrations over 300μg/mL resulted significant cytotoxicity and growth inhibition of the host DH-82 cells (>20%).

**Table 3:** Allopurinol susceptibilities of intracellular amastigote strains. Outcomes are presented as percent inhibition in cultures treated with 300μg/mL allopurinol relative to untreated controls.

Isolate^§^	Average amastigote count per 100 macrophages		Percent inhibition^¶^
	Treated	Untreated	
NT1	47.5±7.8	57.5±13.4	12.3±0.2
NT2	98±0	119.5±6.4	17.9±4.4
NT3	60±0	76±5.7	20.8±5.9
NT4	68.5±0.7	91.5±4.9	25±3.3
NT5	86.5±7.8	109±14.1	20.4±3.2
NT6	68.5±4.9	89±9.9	22.9±3
NT7	76.5±13.4	92.5±16.3	17.3±0
NT8	66.5±4.9	76.5±4.9	13.1±0.8
NT9	95±8.5	123±8.5	22.8±1.6
NT10	99±9.9	136.5±6.4	27.6±3.9
Mean ± STDEV			20.0±4.9*
TR1	82±29.7	82.5±30.4	0.5±0.3
TR2	75±1.4	79.5±3.5	5.6±2.4
TR3	87±5.7	93.5±4.9	7±1.1
TR4	58.5±4.9	66.5±4.9	12.1±0.9
Mean ± STDEV			6.3±4.8

^§^ Parasites isolated from NT (non-treated) or TR (treated relapsed) dogs.

^¶^ Percent Inhibition = [1- (Number of amastigotes in allopurinol treated DH-82 cells / Number of amastigotes in control untreated DH-82 cells)] x 100.

* Represents significant difference between groups (Mann-Whitney test, *P* = 0.002)

No significant correlation was found between the promastigote IC50 and serology results (Spearman’s rho correlation, *r <* 0.17), or treatment length (*r* < 0.58) of dogs from which the respective parasites were isolated.

## Discussion

This is the first detailed report of resistance to allopurinol in *L*. *infantum* parasites isolated from dogs and associated with clinical relapse. All dogs included in this study were seropositive at diagnosis, had typical clinical manifestations of CanL at the time of their initial diagnosis or when they relapsed, and were positive by lymph node culture. The clinical score was able to reliably reflect the clinical status of each group, while serology, complete blood count results, as well as the biochemical indices tested, did not show significant differences between groups that could be beneficial for their clinical characterization. A previous study has demonstrated that an increase in parasite DNA loads in lymph nodes, but not blood, was correlated with the reappearance of clinical signs in relapsing dogs [17]. Our results shows that parasitemia seems to be greatly reduced or absent following treatment, which may be attributed to the effect of allopurinol, causing a total reduction in parasite load, as reported previously [35]. Thus, these results do not indicate that any ancillary test employed in the study is superior to the clinical score in the diagnosis of relapse in leishmaniotic dogs. Although it is widely accepted that dogs treated for visceral leishmaniasis using the currently available drugs frequently remain infected and often relapse [8,13], only a small number of relapse cases have been described so far [14,15,16,17,18], and no characterization of parasite strains from relapsed dogs was made. As opposed to the large volume of evidence on drug resistance in human leishmaniasis, little is known regarding the development of drug resistance in CanL. One study demonstrated that *L*. *infantum* isolates obtained from naturally infected dogs after a therapeutic course of meglumine antimoniate were less susceptible to the drug compared to isolates from the same dogs before treatment [25]. Similar results were also demonstrated in a canine experimental *L*. *infantum* infection model followed by meglumine antimoniate treatment [26]. Differences in susceptibility to meglumine antimoniate among *L*. *infantum* isolates belonging to two different zymodemes from an area lacking drug pressure have also been shown and were hypothesized to represent an inherent resistance to this drug [27]. As part of a larger study on drug susceptibility in humans and dogs, allopurinol susceptibility was evaluated in two *L*. *infantum* isolates from dogs [36]. The IC50 value of an isolate from a non-treated dog was found to be lower compared to an isolate from a dog undergoing treatment. However, the clinical status of the latter dog was not discussed, and no association was made between clinical relapse and decreased drug susceptibility of parasites. We have found a significant 3 to 4 fold increase in allopurinol IC50 for promastigote strains isolated from relapsed dogs (TR) undergoing treatment with this drug compared to non-treated dogs. This increase was evident when testing axenic amastigotes and intracellular amastigotes. Although we did not find a significant correlation between treatment duration and drug IC_50_, the fact that increase in drug resistance was found in parasites isolated from treated dogs suggests that resistance may at least in some cases be caused by selection under drug pressure. Resistance development over time under the pressure of drug treatment has been described for *L*. *donovani* and antimonials [21]. In addition, inherent increased resistance to drugs must also be considered, in view of the considerable variation in IC50 and the % inhibition values demonstrated within each group in all parasite life stages tested. The dissemination of drug resistant parasites may provide another possible explanation for this variation. Cases such as the TR4 isolate, which presented increased drug resistance while being under drug pressure for only short time, may be attributed to either of the above options. Although relapse was associated in this study with parasite drug resistance, a future study with a larger number of relapsed dogs may identify animals with lower IC_50_’s that relapsed due to other potential causes such as concurrent immune-suppressing conditions and neoplasia. By widening the database of dogs and parasites we may better understand the origin of these resistant parasites and take measures to minimize their prevalence. The association found in this study between clinical relapse and resistant parasites may affect current drug selection and treatment regimens. Since no significant difference was found in serology values between the groups and no parasite DNA was detected in the blood of most relapsed dogs, these routine tests are ineffective in discriminating between infection with sensitive and resistant parasites. Relapse in leishmaniotic dogs presents a clinical challenge, since possible effects of deteriorating leishmanial infection such as kidney disease, as well as nonrelated conditions such as other diseases, should be assessed before parasite drug resistance is defined as the cause of relapse. Despite that, our results suggest that allopurinol resistance should be considered, and that an affordable methodology to test the presence of allopurinol resistant *L*. *infantum* in dogs should be available, once clinical relapse is suspected.

In conclusion, this study is the first report of resistance to allopurinol in *L*. *infantum* isolates from dogs with clinical disease relapse. Resistance was verified in three forms of the parasite. Clinical relapse associated with naturally developing resistance to allopurinol is a serious cause for concern considering the zoonotic nature of the disease, and allopurinol's major role in long term treatment of CanL. As canines with uncontrollable *L*. *infantum* infection are highly infectious to sand flies [37], dogs infected with allopurinol resistant strains may pose great risk for infection of naive dogs as well as humans.

## Supporting Information

S1 Table Identifiers and ITS1 sequences GenBank accession numbers for *L*. *infantum* isolates used in the study. A fragment containing leishmanial ITS1 and partial 5.8 rRNA gene was amplified by followed by sequencing and analysis using BLAST (www.ncbi.nlm.nih.gov/BLAST).
(DOCX)

S2 Table Results of clinical and laboratory evaluation of study dogs and respective *Leishmania* isolates. (DOCX)
