# Supplementary material for: Allopurinol Resistance in Leishmania infantum from Dogs with Disease Relapse
Source: PLoS Negl Trop Dis. 2016 Jan 6;10(1):e0004341. doi: 10.1371/journal.pntd.0004341 (PMC4711794; doi:10.1371/journal.pntd.0004341)
Supplement: S1 Table — A fragment containing leishmanial ITS1 and partial 5.8 rRNA gene was amplified by followed by sequencing and analysis using BLAST (www.ncbi.nlm.nih.gov/BLAST). (DOCX) [file pntd.0004341.s001.docx]

**S1 Table**. **Identifiers and ITS1 sequences GenBank accession numbers for *L. infantum* isolates from study dogs**. A fragment containing leishmanial ITS1 and partial 5.8 rRNA gene was amplified followed by sequencing and analysis using BLAST (www.ncbi.nlm.nih.gov-/BLAST).

| **Isolate** | **WHO name** | **LRC number** | **GenBank accession number** |
| --- | --- | --- | --- |
| NT1 | MCAN/IL/2010/NT1 | LRC-L1409 | KM677128 |
| NT2 | MCAN/IL/2011/NT2 | LRC-L1518 | KM677129 |
| NT3 | MCAN/IL/2010/NT3 | LRC-L1476 | KM677130 |
| NT4 | MCAN/IL/2011/NT4 | LRC-L1524 | KM677131 |
| NT5 | MCAN/IL/2011/NT5 | LRC-L1674 | KM677132 |
| NT6 | MCAN/IL/2011/NT6 | LRC-L1532 | KM677133 |
| NT7 | MCAN/IL/2011/NT7 | LRC-L1552 | KM677134 |
| NT8 | MCAN/IL/2011/NT8 | LRC-L1678 | KM677135 |
| NT9 | MCAN/IL/2011/NT9 | LRC-L1556 | KM677136 |
| NT10 | MCAN/IL/2011/NT10 | LRC-L1557 | KM677137 |
| TR1 | MCAN/IL/2010/TR1 | LRC-L1438 | KM677138 |
| TR2 | MCAN/IL/2009/TR2 | LRC-L1401 | KM677139 |
| TR3 | MCAN/IL/2011/TR3 | LRC-L1511 | KM677140 |
| TR4 | MCAN/IL/2011/TR4 | LRC-L1677 | KM677141 |
| TA1 | MCAN/IL/2011/TA1 | LRC-L1551 | KM677142 |
| TA2 | MCAN/IL/2011/TA2 | LRC-L1550 | KM677143 |
| TA3 | MCAN/IL/2011/TA3 | LRC-L1555 | KM677144 |
| TA4 | MCAN/IL/2011/TA4 | LRC-L1673 | KM677145 |
| TA5 | MCAN/IL/2011/TA5 | LRC-L1675 | KM677146 |
