# Supplementary material for: Allopurinol Resistance in Leishmania infantum from Dogs with Disease Relapse
Source: PLoS Negl Trop Dis. 2016 Jan 6;10(1):e0004341. doi: 10.1371/journal.pntd.0004341 (PMC4711794; doi:10.1371/journal.pntd.0004341)
Supplement: S2 Table — (DOCX) [file pntd.0004341.s002.docx]

**S2 Table.**  **Results of clinical and laboratory evaluation of study dogs and respective *Leishmania* isolates**. Dogs are divided into three groups: non-treated (NT), treated relapsed (TR) and treated in remission (TA).

| **Dog** | **General** | | | **Blood count *** | | | | | | **Biochemistry** | | | | | **Molecular tests**** | | | |
| --- | --- | --- | --- | --- | --- | --- | --- | --- | --- | --- | --- | --- | --- | --- | --- | --- | --- | --- |
|  | **Gender** | **Age (y)** | **Allopurinol treatment duration (months)** | **White blood cells (10^9^/L) [6-17)** | **Red blood cells (10^12^/L) [5.5-8.5]** | **Hematocrit (%) [37-55)** | **Platelets (10^9^/L) [200-500]** | **Mean corpuscle volume (fL) [60-77]** | **Mean corpuscle hemoglobin concentration (g/dl) [31-34)** | **Serum albumin (g/dl) [2.6-4)** | **Serum total protein (g/dl) [5.4-7.5)** | **Serum globulin (g/dl) [2.7-4.4]** | **Creatinine (g/dl) [0.5-1.5]** | **Urea (g/dl) [12.6-58.3)** | **ELISA (OD)** | **Blood kDNA PCR** | **Blood kDNA PCR - CT (mean)** | **Blood kDNA PCR - quantitative parasites/uL DNA (mean)** |
|  |  |  |  |  |  |  |  |  |  |  |  |  |  |  |  |  |  |  |
| NT1 | F | 3.0 | - | 8.4 | 4.7 | 30.6 | 215.0 | 65.8 | 34.2 | 4.0 | 8.8 | 4.8 | 1.2 | 30.3 | 1.7 | pos | 20.7 | 58.2 |
| NT2 | M | 8.0 | - | 13.9 | 5.3 | 30.0 | 118.0 | 56.7 | 31.6 | 2.0 | 10.6 | 8.6 | 1.1 | 37.4 | 1.1 | pos | 23.1 | 10.0 |
| NT3 | F | 1.5 | - | 5.2 | 4.6 | 29.0 | 205.0 | 63.1 | 33.6 | 2.0 | 8.9 | 6.9 | 1.1 | 31.0 | 1.6 | pos | 20.3 | 75.2 |
| NT4 | M | 11.0 | - | 8.2 | 4.1 | 28.6 | 226.0 | 70.0 | 31.0 | 2.9 | 7.4 | 4.5 | 3.0 | 150.0 | 1.6 | pos | 31.3 | <1 |
| NT5 | M | 5.0 | - | 4.8 | 4.2 | 28.4 | 167.0 | 67.7 | 35.4 | 2.2 | 9.0 | 6.8 | 0.6 | 15.2 | 1.6 | pos | 27.5 | <1 |
| NT6 | M | 11.0 | - | 43.6 | 4.5 | 29.0 | 469.0 | 64.7 | 33.7 | 2.6 | 6.6 | 4.0 | 0.6 | 39.0 | 1.1 | pos | 20.5 | 63.9 |
| NT7 | M | 11.0 | - | 12.6 | 4.8 | 30.5 | 338.0 | 63.8 | 34.9 | 1.7 | 5.5 | 3.8 | 0.5 | 36.6 | 0.9 | pos | 26.6 | <1 |
| NT8 | M | 2.5 | - | 15.3 | 4.2 | 27.0 | 123.0 | 62.4 | 32.1 | 1.6 | 11.0 | 9.5 | 0.9 | 42.1 | 1.5 | pos | 19.5 | 133.4 |
| NT9 | F | 5.5 | - | 6.9 | 6.0 | 32.8 | 349.0 | 54.7 | 32.6 | 2.4 | 10.2 | 7.8 | 0.8 | 14.5 | 1.8 | pos | 23.9 | 5.8 |
| NT10 | F | 5.0 | - | 8.6 | 3.8 | 22.9 | 758.0 | 60.0 | 30.3 | 2.1 | 8.4 | 6.3 | 1.4 | 75.3 | 1.3 | neg | neg | neg |
| **Mean** |  | **6.4** |  | **12.7** | **4.6** | **28.9** | **296.8** | **62.9** | **32.9** | **2.3** | **8.6** | **6.3** | **1.1** | **47.1** | **1.4** |  |  |  |
| **STDEV** |  | **3.7** |  | **11.4** | **0.6** | **2.6** | **196.1** | **4.7** | **1.7** | **0.7** | **1.8** | **2.0** | **0.7** | **39.9** | **0.3** |  |  |  |
|  |  |  |  |  |  |  |  |  |  |  |  |  |  |  |  |  |  |  |
| TR1 | F | 6.0 | 36 | 7.6 | 3.9 | 22.1 | 329.0 | 57.0 | 36.6 | 2.8 | 7.4 | 4.6 | 2.1 | 56.0 | 0.5 | neg | neg | neg |
| TR2 | F | 8.0 | 36 | 13.9 | 5.3 | 29.1 | 69.0 | 56.0 | 34.7 | 1.9 | 5.5 | 3.6 | 2.0 | 75.6 | 1.3 | pos | 22.8 | 14.0 |
| TR3 | F | 10.0 | 24 | 9.1 | 4.2 | 29.3 | 287.0 | 70.6 | 32.5 | 2.4 | 7.7 | 5.4 | 1.3 | 67.7 | 1.8 | ND | ND | ND |
| TR4 | F | 3.0 | 4 | 37.7 | 3.6 | 26.8 | 139.0 | 74.7 | 33.7 | 0.9 | 4.7 | 3.8 | 0.5 | 72.9 | 1.5 | neg | neg | neg |
| **Mean** |  | **6.8** | **25.0** | **17.1** | **4.2** | **26.8** | **206.0** | **64.6** | **34.4** | **2.0** | **6.3** | **4.3** | **1.5** | **68.1** | **1.3** |  |  |  |
| **STDEV** |  | **3.0** | **15.1** | **14.0** | **0.7** | **3.3** | **122.4** | **9.5** | **1.7** | **0.8** | **1.5** | **0.8** | **0.7** | **8.7** | **0.6** |  |  |  |
|  |  |  |  |  |  |  |  |  |  |  |  |  |  |  |  |  |  |  |
| TA1 | M | 5.0 | 11 | 8.1 | 4.4 | 30.4 | 340.0 | 62.0 | 32.5 | 2.4 | 5.6 | 3.2 | 0.6 | 30.4 | 0.8 | neg | neg | neg |
| TA2 | F | 3.0 | 10 | 7.1 | 4.0 | 25.6 | 218.0 | 57.1 | 33.7 | 2.3 | 6.5 | 4.2 | 0.8 | 45.2 | 1.1 | neg | neg | neg |
| TA3 | M | 11.0 | 4 | 14.2 | 4.3 | 28.3 | 273.0 | 71.3 | 31.5 | 2.5 | 6.5 | 4.0 | 2.9 | 131.5 | 1.3 | neg | neg | neg |
| TA4 | F | 6.0 | 8 | 12.3 | 5.2 | 30.2 | 291.0 | 65.6 | 33.1 | 2.2 | 6.9 | 4.7 | 0.9 | 34.4 | 1.3 | neg | neg | neg |
| TA5 | F | 6.0 | 19 | 7.2 | 3.4 | 26.3 | 320.0 | 68.1 | 32.9 | 2.1 | 7.0 | 4.9 | 0.5 | 22.9 | 1.0 | neg | neg | neg |
| **Mean** |  | **6.2** | **10.3** | **9.8** | **4.3** | **28.2** | **288.4** | **64.8** | **32.7** | 2.3 | 6.4 | 4.0 | 1.3 | 60.4 | **1.1** |  |  |  |
| **STDEV** |  | **2.9** | **5.6** | **3.3** | **0.7** | **2.2** | **47.1** | **5.5** | **0.8** | **0.1** | **0.6** | **0.7** | **1.0** | **44.7** | **0.2** |  |  |  |

* For blood count and biochemical indices - normal reference range in square brackets

** ND - not determined, pos - Positive, neg – Negative

| **Dog** | **Clinical scoring - at diagnosis** | | | | | | | | | **Clinical scoring - at enrollment*** | | | | | | | | | **Allopurinol sensitivity**** | | |
| --- | --- | --- | --- | --- | --- | --- | --- | --- | --- | --- | --- | --- | --- | --- | --- | --- | --- | --- | --- | --- | --- |
|  | **Lymph node size**  **(0-1)** | **Skin lesions (0-2)** | **Weight loss**  **(0-2)** | **Hemorrhagic diarrhea (0-1)** | **Hyperthermia (0-1)** | **Ocular lesions (0-1)** | **Orchitis (0-1)** | **Hematuria (0-1)** | **Total score (out of 10)** | **Lymph node size**  **(0-1)** | **Skin lesions (0-2)** | **Weight loss**  **(0-2)** | **Hemorrhagic diarrhea (0-1)** | **Hyperthermia (0-1)** | **Ocular lesions (0-1)** | **Orchitis (0-1)** | **Hematuria (0-1)** | **Total score (out of 10)** | **Promast IC50 (µg/mL)** | **Axenic amast IC50 (µg/mL)** | **Intra-cellular amast (% inhibition)** |
|  |  |  |  |  |  |  |  |  |  |  |  |  |  |  |  |  |  |  |  |  |  |
| NT1 |  |  |  |  |  |  |  |  |  | 1 | 1 | 0 | 0 | 0 | 1 | NR | 0 | 3 | 552.7 | 817.0 | 12.3 |
| NT2 |  |  |  |  |  |  |  |  |  | 1 | 2 | 2 | 0 | 1 | 1 | 0 | 0 | 7 | 332.7 | 623.0 | 17.9 |
| NT3 |  |  |  |  |  |  |  |  |  | 1 | 1 | 1 | 0 | 0 | 0 | NR | 0 | 3 | 188.3 | 648.5 | 20.8 |
| NT4 |  |  |  |  |  |  |  |  |  | 1 | 1 | 2 | 0 | 1 | 1 | 0 | 0 | 6 | 104.5 | 738.0 | 25.0 |
| NT5 |  |  |  |  |  |  |  |  |  | 1 | 2 | 1 | 0 | 0 | 0 | 0 | 0 | 4 | 93.3 | 546.5 | 20.4 |
| NT6 |  |  |  |  |  |  |  |  |  | 1 | 1 | 2 | 0 | 0 | 0 | 0 | 0 | 4 | 80.2 | 817.5 | 22.9 |
| NT7 |  |  |  |  |  |  |  |  |  | 1 | 2 | 2 | 0 | 0 | 1 | 0 | 0 | 6 | 106.6 | 794.0 | 17.3 |
| NT8 |  |  |  |  |  |  |  |  |  | 1 | 2 | 2 | 0 | 1 | 1 | 0 | 0 | 7 | 181.6 | 705.5 | 13.1 |
| NT9 |  |  |  |  |  |  |  |  |  | 1 | 1 | 2 | 0 | 1 | 0 | NR | 0 | 5 | 216.4 | 598.5 | 22.8 |
| NT10 |  |  |  |  |  |  |  |  |  | 1 | 1 | 1 | 0 | 1 | 1 | NR | 0 | 5 | 146.1 | 421.5 | 27.6 |
| **Mean** |  |  |  |  |  |  |  |  |  |  |  |  |  |  |  |  | 0 | **5.0** | **200.2** | **671.0** | **20.0** |
| **STDEV** |  |  |  |  |  |  |  |  |  |  |  |  |  |  |  |  |  | **1.5** | **145.0** | **128.8** | **4.9** |
|  |  |  |  |  |  |  |  |  |  |  |  |  |  |  |  |  |  |  |  |  |  |
| TR1 | 1 | 1 | 1 | 0 | 0 | 1 | NR | 0 | 4 | 1 | 1 | 2 | 0 | 1 | 0 | NR | 0 | 5 | 1520.0 | 1833.5 | 0.5 |
| TR2 | 1 | 2 | 2 | 0 | 1 | 0 | NR | 0 | 6 | 1 | 2 | 2 | 0 | 1 | 1 | NR | 0 | 7 | 996.3 | 2127.0 | 5.6 |
| TR3 | 1 | 1 | 2 | 0 | 0 | 0 | NR | 0 | 4 | 1 | 2 | 2 | 0 | 0 | 0 | NR | 0 | 5 | 703.6 | 1550.0 | 7.0 |
| TR4 | 1 | 1 | 1 | 0 | 0 | 1 | NR | 0 | 4 | 1 | 2 | 1 | 0 | 1 | 1 | NR | 0 | 6 | 762.4 | 1200.5 | 12.1 |
| **Mean** |  |  |  |  |  |  |  |  | **4.5** |  |  |  |  |  |  |  |  | **5.8** | **995.5** | **1677.8** | **6.3** |
| **STDEV** |  |  |  |  |  |  |  |  | **1.0** |  |  |  |  |  |  |  |  | **1.0** | **371.8** | **395.9** | **4.8** |
|  |  |  |  |  |  |  |  |  |  |  |  |  |  |  |  |  |  |  |  |  |  |
| TA1 | 1 | 1 | 2 | 0 | 1 | 0 | 0 | 0 | 5 | 1 | 0 | 0 | 0 | 0 | 0 | 0 | 0 | 1 | 120.9 |  |  |
| TA2 | 1 | 2 | 2 | 0 | 1 | 0 | NR | 0 | 6 | 0 | 0 | 1 | 0 | 0 | 0 | NR | 0 | 1 | 293.1 |  |  |
| TA3 | 1 | 2 | 1 | 0 | 0 | 1 | 0 | 0 | 5 | 0 | 0 | 1 | 0 | 0 | 0 | 0 | 0 | 1 | 62.4 |  |  |
| TA4 | 1 | 1 | 1 | 0 | 0 | 0 | NR | 0 | 3 | 1 | 0 | 0 | 0 | 0 | 0 | NR | 0 | 1 | 414.7 |  |  |
| TA5 | 1 | 2 | 1 | 0 | 0 | 1 | NR | 0 | 5 | 0 | 1 | 0 | 0 | 0 | 0 | NR | 0 | 1 | 447.5 |  |  |
| **Mean** |  |  |  |  |  |  |  |  | **4.8** |  |  |  |  |  |  |  |  | **1.0** | **267.7** |  |  |
| **STDEV** |  |  |  |  |  |  |  |  | **1.1** |  |  |  |  |  |  |  |  | **0.0** | **171.9** |  |  |

**S2 Table.**  **- continued**

*NR - not relevant

**Promast – promastigotes, amast - amastigotes
